# Low picomolar, instrument-free visual detection of mercury and silver ions using low-cost programmable nanoprobes

**DOI:** 10.1039/c6sc03444f

**Published:** 2016-10-29

**Authors:** Muhit Rana, Mustafa Balcioglu, Neil M. Robertson, Mustafa Salih Hizir, Sumeyra Yumak, Mehmet V. Yigit

**Affiliations:** a Department of Chemistry , University at Albany , State University of New York , 1400 Washington Avenue , Albany , New York 12222 , USA . Email: myigit@albany.edu ; Tel: +1-518-442-3002; b The RNA Institute , University at Albany , State University of New York , 1400 Washington Avenue , Albany , New York 12222 , USA; c Department of Science , City University of New York , BMCC , 199 Chambers Street , New York , 10007 NY , USA

## Abstract

The EPA's recommended maximum allowable level of inorganic mercury in drinking water is 2 ppb (10 nM). To our knowledge, the most sensitive colorimetric mercury sensor reported to date has a limit of detection (LOD) of 800 pM. Here, we report an instrument-free and highly practical colorimetric methodology, which enables detection of as low as 2 ppt (10 pM) of mercury and/or silver ions with the naked eye using a gold nanoprobe. Synthesis of the nanoprobe costs less than $1.42, which is enough to perform 200 tests in a microplate; less than a penny for each test. We have demonstrated the detection of inorganic mercury from water, soil and urine samples. The assay takes about four hours and the color change is observed within minutes after the addition of the last required element of the assay. The nanoprobe is highly programmable which allows for the detection of mercury and/or silver ions separately or simultaneously by changing only a single parameter of the assay. This highly sensitive approach for the visual detection relies on the combination of the signal amplification features of the hybridization chain reaction with the plasmonic properties of the gold nanoparticles. Considering that heavy metal ion contamination of natural resources is a major challenge and routine environmental monitoring is needed, yet time-consuming, this colorimetric approach may be instrumental for on-site heavy metal ion detection. Since the color transition can be measured in a variety of formats including using the naked eye, a simple UV-Vis spectrophotometer, or recording using mobile phone apps for future directions, our cost-efficient assay and method have the potential to be translated into the field.

## Abstract 2

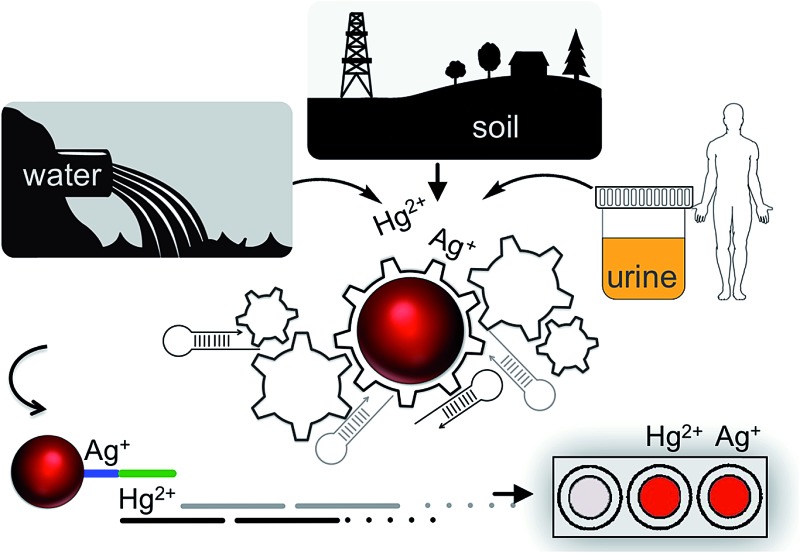
A single gold nanoprobe can be programmed for low picomolar visual detection of inorganic mercury and/or silver in water, soil or urine samples.

Environmental contamination by heavy metal ions is a serious global concern.^1,2^ Mercury is one of the most toxic heavy metals which accumulates biologically and causes various health problems including brain damage, kidney failure, and various motion disorders.^3–5^ Since even small quantities of mercury can be highly toxic it is critical to develop highly sensitive methodologies for its detection.^6^ Mercury can exist in various forms: organic, inorganic or elemental where each species can display a different level of toxicity.^2^ While the classification of each species is important for taking proper precautions, the detection of inorganic mercury, which is considered to be the precursor of organic or elemental mercury, has captured particular attention.^7–11^ Though not considered as toxic as mercury, inorganic silver is another highly toxic metal ion which can also result in a variety of human health problems and has been found to be extremely toxic to aquatic animals.^12–15^ The amount of mercury and silver can be found above its tolerable contamination level in water and soil samples, and can be ingested through drinking water or *via* the food chain. Therefore, it is critical to monitor natural sources for potential heavy metal contamination.^16–18^ Particularly, the recent lead contamination incident in the drinking water supply of Flint, Michigan (USA), followed by numerous poisoning cases and fatalities, reminded us, once again, of the significance of heavy metal ion monitoring in our natural resources.^19,20^

According to the U. S. Environmental Protection Agency (EPA) the recommended maximum level of inorganic mercury in the drinking water is 2 ppb (10 nM)^21^ and the tissue-based water from fish is 3 ppm (1.5 μM).^22^ The Agency for Toxic Substances and Disease Registry (ATSDR) of the U. S. Department of Health and Human Service sets the highest mercury concentration as 625 ppb for normal soil.^23^ The EPA recently reported a four-year long study on different species of fish from the 76 559 lakes in 48 states of the USA, of them, 49% of the sampled population of lakes (36 422 lakes) exceeded the EPA recommended concentration.^22^ These results urged us to develop practical sensors that can provide on-site accurate results in shorter time frames. Such an enterprise will enable us to take quicker actions to avoid mercury poisoning through the consumption of water or food supplies from contaminated resources. Besides the detection in the natural resources, industrial sites or agricultural products it is also critical and thus required to monitor mercury content in human bodily fluids to determine the level of exposure. For instance, the New York State (NYS) sanitary code requires healthcare providers to report blood or urine mercury levels to the NYS Department of Health when mercury concentration is at or above 5 ppb in blood and 20 ppb in urine.^24^ These standards, along with many other studies, stress the importance of and need for easy-to-use, fast and cost-efficient mercury sensors for environmental and biological screenings.^25^

At present, there are various methods available for the detection of heavy metal ions: atomic absorption spectroscopy,^26^ inductively coupled plasma mass spectrometry (ICP-MS),^27^ cold vapor atomic fluorescence spectroscopy (CV-AFS)^28^ and atomic fluorescence spectroscopy.^29^ Though these well-established methods are very sensitive and provide accurate results, they require large sample volumes, time-consuming sample processing prior to the detection and trained personnel for the operation of sophisticated instrumentation. Moreover, the instruments needed for these detection approaches are costly and not portable, and therefore are not suitable for on-site quick screening. There have been numerous attempts to develop methodologies for mercury detection using fluorescence, Raman or absorbance spectroscopies as an alternative to the existing methodologies.^30–33^ Among those the most attractive ones for on-site applications are the ones with colorimetric read-out signals, however most of these approaches suffer from low sensitivity or selectivity.^32,34–36^ Here we combined the programmable features of DNA nanotechnology with the plasmonic properties of metallic nanoparticles for the development of one of the most sensitive inorganic mercury and/or silver sensors using specific pyrimidine interactions in DNA duplexes as described below.^37–42^

Studies have shown that thymine–thymine (T–T) and cytosine–cytosine (C–C) base pairs are very selective for capturing Hg^2+^ and Ag^+^ and form T–Hg^2+^–T and C–Ag^+^–C bridges in DNA duplexes, respectively.^43–45^ These pyrimidine base pairings with Hg^2+^ and Ag^+^, which are stronger than typical Watson–Crick base-pairings, are central for our reprogrammable and selective detection scenario described herein. In this study, the pyrimidine pairing is followed by a hybridization chain reaction (HCR) for the amplification of the colorimetric signal readout.

The hybridization chain reaction (HCR) is an enzyme-free DNA polymerization process where two metastable species of DNA hairpins (H1 & H2) coexist in solution without binding to each other (Fig. 1a, step 1).^42,46,47^ However, in the presence of a specific short single stranded (ss) DNA (initiator = I) molecule the hairpins are activated and self-assemble to form a long double stranded (ds) DNA polymer. Briefly, the HCR process is as follows: the initiator (I) binds and opens the first hairpin (H1, Fig. 1a, step 2) which immediately binds and opens the second hairpin (H2, Fig. 1a, step 3). The opening of H2 forms a dsDNA with a sticky end and induces the activation of another H1 initiating the HCR, (Fig. 1a, step 4). As a result, two hairpin pairs assemble into a long DNA polymer, which was unable to happen in the absence of the initiator strand. Because of its extraordinary signal amplification properties, this enzyme-free DNA polymerization step is essential for achieving the outstanding sensitivity observed in our approach.^40–42^

**Fig. 1:**
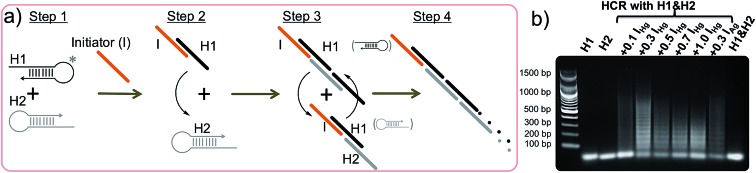
Hybridization chain reaction triggered by initiators. (a) Schematic illustration of the hybridization chain reaction (HCR) triggered by an initiator. (b) Gel electrophoresis data show the polymerization of the H1 & H2 hairpin pair with different amounts of initiator.

## Results and discussion

The studies were performed using two different initiators (I_Hg_ and I_Ag_), each one used for programming the sensor template for a different metal ion detection; I_Hg_ for mercury or I_Ag_ for silver ions. Prior to the detection of the metal ions, the HCR was validated using gel electrophoresis using various amounts of initiator (I_Hg_) with respect to the H1 & H2 hairpin pair (0.1, 0.3, 0.5, 0.7 and 1.0 folds), Fig. 1b. The results obtained with a number of different ratios illustrates the formation of large smeared DNA bands by the activation of the hairpins. These large DNA bands were not observed in the absence of initiators using H1 & H2 only (last lane, Fig. 1b), H1 only (lane 1) or H2 only (lane 2). After confirming the affinity of HCR in the presence of the designed initiator strands (I_Hg_ and I_Ag_) and both hairpins (H1 & H2), we used these components for the detection studies illustrated below.

**Fig. 2:**
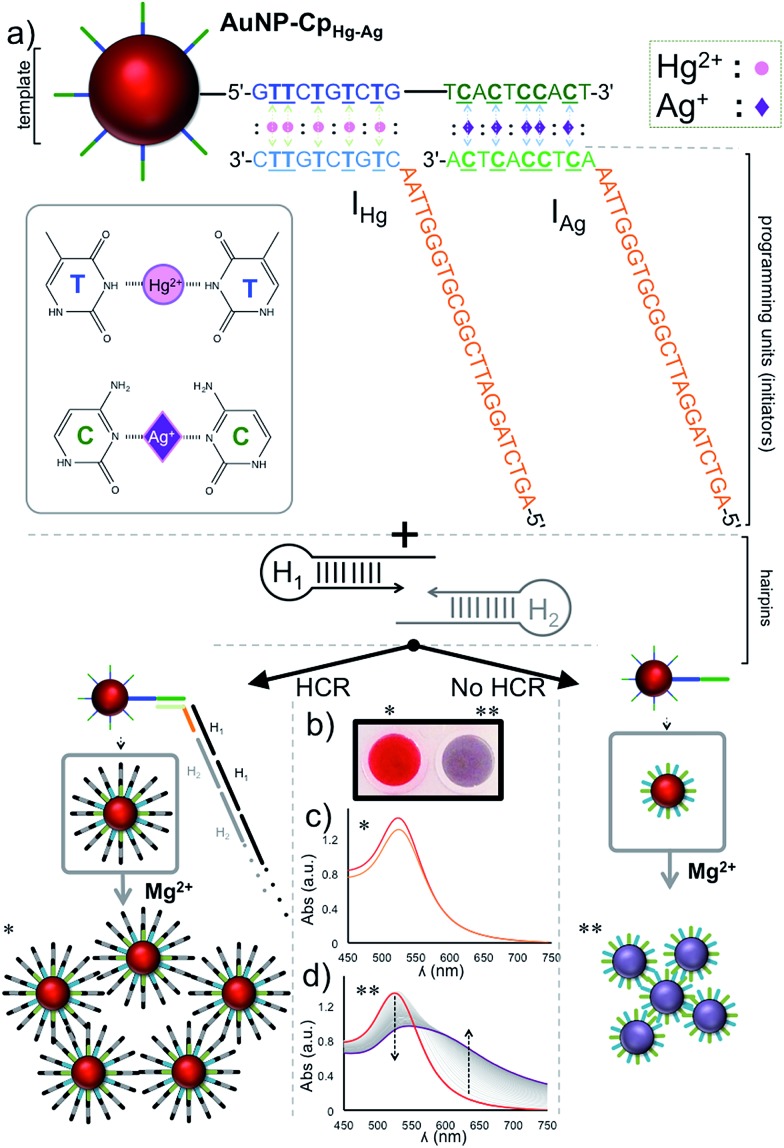
Schematic illustration of the reprogrammable detection using the nanoprobe and the HCR. The gold nanoprobe (AuNP–Cp_Hg–Ag_) is prepared by functionalization of AuNPs with the Cp_Hg–Ag_ capture probe. (a) The Hg^2+^ connects the AuNP–Cp_Hg–Ag_ with the I_Hg_ through the underlined five T bases, located in the dark blue region of Cp_Hg–Ag_ and the light blue region at the 3′ end of I_Hg_, through T–Hg^2+^–T bridges whereas Ag^+^ links the Cp_Hg–Ag_ with I_Ag_ *via* the C–Ag^+^–C pairing. The resulting sandwiched assembly has a universal (orange colored) DNA sticky end which later triggers the hybridization chain reaction (HCR) by activating the H1 & H2 hairpins. The resulting DNA polymers protect the AuNPs from Mg^2+^ induced aggregation (lower left panel) however in the absence of the target metal ions the AuNPs are subject to Mg^2+^ induced aggregation (lower right panel). The change in the aggregation state of the AuNPs was used for validating the presence of Hg^2+^ and characterized both in the (b and c) presence and (b and d) absence of Hg^2+^ and was identified both spectroscopically and visually. Note: the slight decrease in the 520 band in the orange spectrum in (c) is due to a dilution factor introduced by the addition of stock solution Mg(NO_3_)_2_.

Fig. 2a illustrates the schematics of the programmable and visual detection of the Hg^2+^ and Ag^+^ by combining the amplification feature of the HCR with the surface plasmon properties of the gold nanoparticles. First, the 20 nt-long thiolated capture probe (Cp_Hg–Ag_) was immobilized on a gold nanoparticle forming the AuNP–Cp_Hg–Ag_ nanoprobe. The first half of the Cp_Hg–Ag_, from the 5′ end, was designed for the detection of Hg^2+^ whereas the second half was used for the detection of Ag^+^ (Fig. 2a). Separately, two different short single stranded initiators (I_Hg_ for Hg^2+^ and I_Ag_ for Ag^+^) were designed for programming the nanoprobe for individual or simultaneous detection of the metal ions. The 10 nts at the 3′ ends (light colored blue region in I_Hg_ and green region in I_Ag_, Fig. 2a) of the initiator strands serve as the target-recognition regions, while the remaining 24 nt-long regions (orange colored, Fig. 2a) are able to trigger the HCR and are identical for both initiators. The visual detection assay begins as follows; the underlined five T bases in the first half of the Cp_Hg–Ag_ (dark blue region) and in the 3′ tail of I_Hg_ (light blue region) are bridged in the presence of Hg^2+^ forming T–Hg^2+^–T pairs whereas the five C pairs in the second half of the C_Hg–Ag_ (dark green region) and in the 3′ end of I_Ag_ (light green region) are paired with Ag^+^ forming and C–Ag^+^–C bridges (Fig. 2a inset). After the initiators bind to the AuNP–Cp_Hg–Ag_, the resulting purified nanoprobe assembly initiates the HCR by opening the first hairpin, H1 (black colored hairpin, Fig. 2a), in the presence of the H1 & H2 hairpins. The opening of H1 triggers the opening of H2 (gray colored hairpin, Fig. 2a) and starts the HCR. As a result, the surface of the nanoprobe is covered with the DNA polymers composed of alternating H1 & H2 strands illustrated as black and gray stripes on AuNP–Cp_Hg–Ag_ (Fig. 2a * in the lower left panel). This assembly occurs only in the presence of a specific metal ion (Hg^2+^ or Ag^+^) with its initiator pair, increasing the hydrodynamic size of the AuNP–Cp_Hg–Ag_ from 16.9 ± 0.7 nm to 153.6 ± 20.3 nm after the polymerization on the nanoprobe surface.

Finally, in order to monitor the effect of the HCR-induced DNA polymerization on the nanoparticle surface, and therefore evaluate the detection of Hg^2+^ or Ag^+^, Mg^2+^ ion salt (∼55 mM nitrate salt) was added to the resulting nanoparticle assembly to aggregate the gold nanoparticles.^47^ While AuNPs with low and/or short DNA coating density can aggregate under this high salt condition, DNA polymers anchored on the surface of the nanoparticles can protect the nanoparticles from aggregation by shielding this charge effect. To demonstrate the working principle of the assay the studies were performed separately for both metal ions (Fig. 2b–d, shown only for Hg^2+^ in the figure). As anticipated (Fig. 2a ** in the lower right panel), an immediate salt-induced AuNP aggregation was observed in the form of a red-purple-gray color transition (Fig. 2b) and shift in the surface plasmon band at ∼520 nm (Fig. 2d) in the absence of the target metal ion. On the other hand, in the presence of the target metal ion the DNA polymers on the gold surface protected the nanoparticles from Mg^2+^-induced aggregation, which was recorded by no change in the spectrum of the gold nanoparticles (Fig. 2c) and a retained red color of the colloidal suspension (Fig. 2b).

**Fig. 3:**
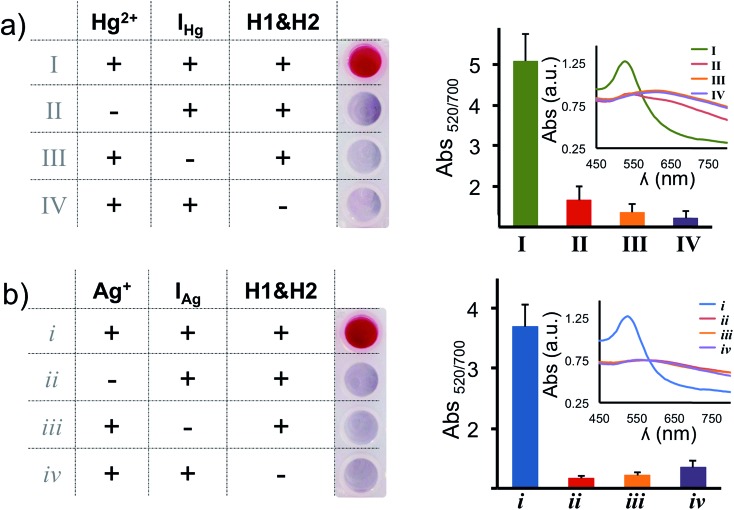
Absence test: validating the necessity of the programming and amplification components for the detection of each target metal ion. H1 & H2 hairpins and initiators strands (I_Hg_ or I_Ag_) are essential components for the programmable detection of each subtype. The necessity of each component for the visual and spectroscopic detection of (a) Hg^2+^ and (b) Ag^+^ is validated in the absence of target metal ions (II and ii), initiators (III and iii) and hairpins (IV and iv), respectively.

In our assay the H1 & H2 hairpins are the building blocks of the DNA polymers on the nanoprobe surface, the initiator strands (I_Hg_ or I_Ag_) are necessary to initiate the HCR and target metal ions (Hg^2+^ and Ag^+^) are required to anchor the HCR-induced DNA polymers on the nanoparticle surface. In order to validate the necessity of each component, we have performed absence tests for each target metal ion (Hg^2+^ and Ag^+^), separately. As seen in Fig. 3a and b, in the absence of any of the components (initiator, H1 & H2 hairpins or target metal ion), salt-induced AuNP aggregation was observed which was recorded as a color transition and a change in the absorbance spectra. On the other hand, only the nanoparticles with essential components for the HCR retained the original spectral information and color of the assay (Fig. 3a and b).

**Fig. 4:**
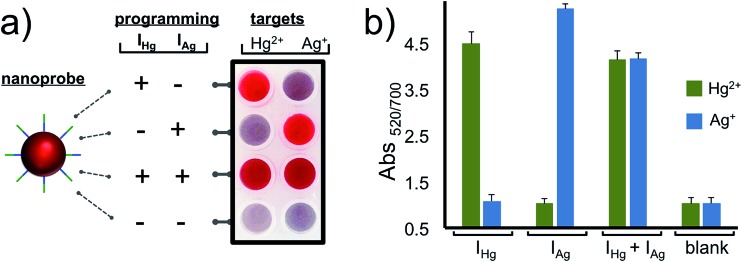
Reprogrammable detection of Hg^2+^ or Ag^+^ in different combinations. Programming the same nanoprobe (AuNP–Cp_Hg–Ag_) for the simultaneous or individual detection of Hg^2+^ and Ag^+^ in four different combinations. Only in the presence of a target metal ion does the programmed assay allow (a) visual and (b) spectroscopic detection of Hg^2+^ and/or Ag^+^.

After characterizing our system and validating it for the detection of Hg^2+^ and Ag^+^ separately, we next demonstrated the programmability feature of our nanoprobe for each target in a 2 × 4 array as illustrated in Fig. 4. The AuNP–Cp_Hg–Ag_ was programmed for the detection of Hg^2+^, but not Ag^+^, when I_Hg_ was used as an initiator strand (programming unit). This was observed as a retained red color with Hg^2+^ and a red-purple-gray color transition with Ag^+^. On the other hand, using I_Ag_ instead of I_Hg_ programs the same AuNP–C_Hg–Ag_ for Ag^+^, but not Hg^2+^, (second row in Fig. 4a and b). In order to use the same nanoprobe for the simultaneous detection of Hg^2+^ and Ag^+^, both initiators were included in the assay. The observed retained red color with both metal ions demonstrates that the same nanoprobe can be programmed for the simultaneous detection of both ions. Finally, in order to illustrate that the programmability of the nanoprobe depends on the initiators, studies were carried out in the absence of initiators. In the presence of any of the metal ions (Hg^2+^ or Ag^+^), nanoprobes aggregated (last row Fig. 4a and b) which confirms the necessity of the initiator strands for programming the nanoprobe for the detection. The results of the 2 × 4 array tests in Fig. 4 overall suggest that the same gold nanoprobe (AuNP–Cp_Hg–Ag_) offers visual and spectroscopic detection of Hg^2+^ and Ag^+^ in all four possible combinations which was achieved by only changing the compositions of the initiator strands in the assay while keeping every other parameter unchanged. The system is highly selective to the programmed settings with no false-positive or false-negative results.

**Fig. 5:**
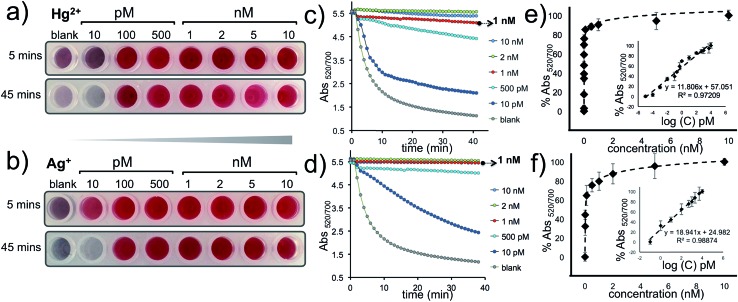
Detection of different concentrations of Hg^2+^ or Ag^+^. The sensitivity of the nanoprobe was evaluated visually (a and b) and spectroscopically by monitoring the aggregation rate (Abs_520/700_ *vs.* time) (c and d) and degree (Abs_520/700_ *vs.* metal ion concentration) for Hg^2+^ and Ag^+^ (e and f).

The approach described here has a remarkable signal amplification feature because the intercalation of five Hg^2+^ or Ag^+^ ions can trigger the HCR and anchor a DNA polymer; sized 1000 bps or longer (Fig. 1b); on the AuNP–Cp_Hg–Ag_ surface that can protect the nanoprobes from a salt-induced color transition. Furthermore, since a single AuNP–Cp_Hg–Ag_ is conjugated to hundreds of capturing probes, this protection is far more pronounced with multiple polymerization sites. The visual sensitivity of our system was tested using 10, 100 and 500 pM and 1, 2, 5 and 10 nM of Hg^2+^ and Ag^+^, separately. The results demonstrate that the assay undergoes a color transition within minutes in the absence of the target metal ion (blank, reference well) and as low as 10 pM (0.4 fmol) of Hg^2+^ or Ag^+^ can be differentiated with the naked eye (first row in Fig. 5a and b). After the color transition has stabilized, the difference becomes exceptionally obvious, about an hour after the addition of Mg^2+^, and as low as 100 pM of Hg^2+^ or Ag^+^ can be visually detected. Additionally, the studies performed by monitoring the aggregation rate (Fig. 5c and d) and degree (Fig. 5e and f), obtained by recording Abs_520/700_ with various concentrations of Hg^2+^ and Ag^+^, illustrate the sensitivity of the assay spectroscopically.

**Fig. 6:**
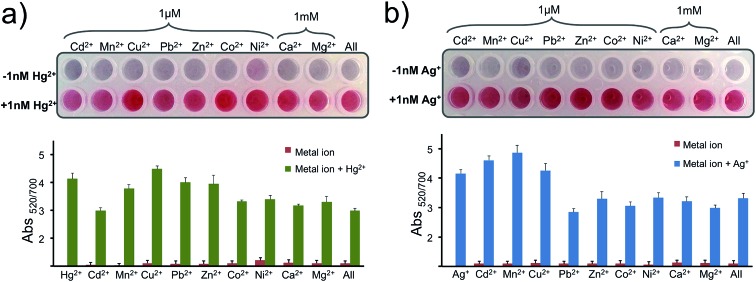
Selectivity for Hg^2+^ or Ag^+^. The selectivity of the assay for (a) Hg^2+^ or (b) Ag^+^ is evaluated by testing the nanoprobe with Cd^2+^, Mn^2+^, Cu^2+^, Pb^2+^, Zn^2+^, Co^2+^, Ni^2+^, Ca^2+^, Mg^2+^ and a soup of all metal ions (last well), where the Ca^2+^ and Mg^2+^ concentrations were chosen as 1 mM and the remaining metal ions' concentrations were 1 μM. In the second row of the assay the studies were performed after the addition of 1 nM of (a) Hg^2+^ or (b) Ag^+^ into each metal ion mixture. Abs_520/700_ was recorded for each well 1 h after the addition of Mg(NO_3_)_2_. Experiments were performed in triplicate.

Later the selectivity of the assay for Hg^2+^ and Ag^+^ was determined using 1 μM of Cd^2+^, Mn^2+^, Cu^2+^, Pb^2+^, Zn^2+^, Co^2+^, Ni^2+^ and 1 mM of Ca^2+^ and Mg^2+^. As seen in Fig. 6a and b, any of these metal ions or a cocktail prepared by combining all of these metal ions in a single well gave a negative result as predicted. With only the addition of 1 nM of Hg^2+^ (Fig. 6a) or Ag^+^ (Fig. 6b) in each well, including the last well with the metal soup, were the nanoprobes protected from aggregating and gave a positive signal. The results overall indicate that the nanoprobe is highly selective to the programmed metal ion and can recognize the target ion selectively in a complex metal ion soup.

**Fig. 7:**
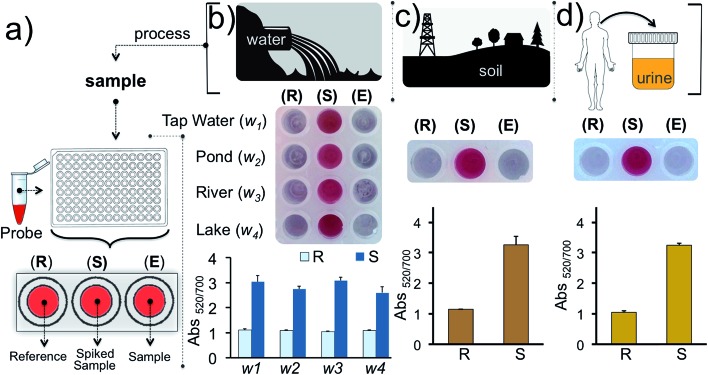
Detection of Hg^2+^ in water, soil and urine samples. (a) Schematic illustration of the detection from different sources using the gold nanoprobe array. Well E is used to monitor the Hg^2+^ in real samples while well S is used to monitor the Hg^2+^ in samples spiked with the 1 nM of Hg^2+^. The reference well (R) contains the nanoprobe tested in reaction buffer and is assessed for comparing the results obtained in well E and S. Experiments were carried out using (b) water samples collected from different sources in the region and (c) soil, and (d) urine. Experiments were performed in triplicate.

Finally, we tested our assay for inorganic mercury detection in real water and soil samples. The array (Fig. 7a) was prepared as follows: experimental well (E) was used to monitor Hg^2+^ in the real sample, well S was used to detect Hg^2+^ in the real sample spiked with 1 nM of Hg^2+^ and a reaction buffer prepared from ultrapure water was used for the reference well (R). The water samples (w_1_, w_2_, w_3_ and w_4_) were obtained from tap water, pond water, river water and lake water, respectively, near regional resources. The results illustrate that the detection of inorganic mercury in real water samples can be performed using this colorimetric methodology (Fig. 7b). Since detection of mercury is important biologically, industrially and agriculturally we also demonstrated the detection of Hg^2+^ in soil and urine samples which were initially spiked with 1 nM of Hg^2+^(Fig. 7c–d). As predicted the samples spiked with 1 nM inorganic mercury retained the nanoprobe's original color whereas in the absence of the target ion a clear color transition was observed. The results overall demonstrate that our assay is capable of detecting inorganic mercury contamination in different environmental or biological matrices.

Though we only focused on using 13 nm sized gold nanoparticles for this study, the performance of the sensor could be further improved by using different sizes and shapes of gold nanoparticles. The surface coverage and the size of the HCR-product on the nanoparticle surface can be increased with bigger nanoparticles, which could, in turn, offer greater sensitivity.

The thymine–thymine (T–T) and cytosine–cytosine (C–C) base pairs in the DNA designs are very specific to Hg^2+^ and Ag^+^, respectively, however a small- or macro-molecule that has a binding affinity to either one of the metal ions could interfere with the sensor's performance. This could be circumvented by either nitrification or a UV digestion procedure prior to detection.^48,49^

## Conclusions

In this study we developed an instrument-free, ultrasensitive, cost-efficient and colorimetric assay, which offers visual detection of as low as 10 pM of inorganic mercury and silver. To our knowledge this is the most sensitive instrument-free detection of each metal ion. We have used our assay for the detection of metal ions in complex metal ion cocktails, water, soil and urine samples. The assay can be used for the individual or simultaneous detection of each metal ion. The assay utilizes the unique pyrimidine–pyrimidine base pairing for specificity, the hybridization chain reaction for sensitivity and programmability, and the plasmonic properties of gold nanoparticles for visual detection. The components of the nanoprobe are inexpensive and easy to obtain or synthesize which makes it ideal for the development of practical sensors for on-site detection. A single test costs less than a penny per well of a 384-well microplate. Measurement of the color change in the assay can be achieved in a variety of formats including by naked eye, UV-Vis spectroscopy and in the future possibly using mobile phone apps. Considering that the heavy metal ion contamination of natural resources is a major concern, this highly practical colorimetric approach could be ideal for on-site heavy metal ion detection not only for resource-limited areas but also for developed industrial sites.

## Materials and methods

### Materials

All oligonucleotide sequences were purchased from Integrated DNA Technologies (IDT), USA with the following sequence information.

#### Thiolated capture strands

(1) Cp_Hg–Ag_:

5′-/5ThioMC6-D/GTTCTGTCTGTCACTCCACT-3′

#### Initiators

(2) I_Hg_:

5′-AGTCTAGGATTCGGCGTGGGTTAACTGTCTGTTC-3′

(3) I_Ag_:

5′-AGTCTAGGATTCGGCGTGGGTTAAACTCCACTCA-3′

#### Hairpins

(4) H1:

5′-TTAACCCACGCCGAATCCTAGACTCAAAGTAGTCTAGGATTCGGCGTG-3′

(5) H2:

5′-AGTCTAGGATTCGGCGTGGGTTAACACGCCGAATCCTAGACTACTTTG-3′

 

Ultrapure RNase-free water was used in all studies. Ethidium bromide (EthBr), Laemmli loading dye, and certified genetic quality tested DNA grade agarose were purchased from Bio-Rad (Hercules, CA, USA). 100 bp DNA ladder was purchased from New England BioLabs, Inc. (Ipswich, MA, USA) for gel electrophoresis studies. Mercury(ii) perchlorate trihydrate (Hg(ClO_4_)_2_·3H_2_O) was purchased from Alfa Aesar and silver nitrate (AgNO_3_) was purchased from Sigma Aldrich. All remaining inorganic metal ions (nitrate salts) were bought from Acros Organics or Thermo Fischer Scientific, NJ, USA. All other reagents were purchased from Sigma-Aldrich, St. Louis, MO 63103, USA.

### Methods

#### Gel electrophoresis

Stock solutions of oligonucleotides were prepared using nuclease-free water. 100 μL of 2 μM H1 and H2 solutions in 50 mM of sodium phosphate buffer (50 mM Na_2_HPO_4_/0.5 M NaNO_3_, pH 7.5) were heated separately to 95 °C in 2.0 mL clear microtubes for 3 min. The solutions were snap-cooled on ice for 3 min and incubated at room temperature (RT) for 2 h before use. The H1 and H2 were mixed (1 : 1) to a final concentration of 1 μM and incubated with 0, 100 nM, 300 nM, 500 nM, 700 nM, and 1000 nM of initiator strands (I_Hg_ or I_Ag_) at 4 °C for 4 hours. The control experiments were performed using H1 alone, H2 alone and H1 & H2 (1 : 1) without the addition of initiator. Briefly, 15 μL of each product and 10 μL of 6× loading dye were mixed in a PCR tube and loaded in the gel. A 1% agarose gel was used in the gel electrophoresis studies and was prepared by heating 1 g of agarose in 100 mL of freshly prepared 1× sodium borate buffer, pH 8.5, for 45 s using a microwave. 10 μL of 10 mg mL^–1^ EthBr was added to the gel solution before polymerization. 1× sodium borate buffer was used as the running buffer and an additional 10 μL of 10 μg mL^–1^ of EthBr was added to the buffer before running the gel. The electrophoresis was performed for 60 min at 4 °C and 100 V. The gels were visualized using a Bio-Rad ChemiDoc™ XRS Imaging System with Quantity One 4.6.1 software.

#### Nanoprobe preparation

Gold nanoparticles (AuNPs) were prepared using the standard citrate reduction method. Briefly, 2 mL of 50 mM HAuCl_4_ was added into 98 mL of boiling DI water in an Erlenmeyer flask. 10 mL of 38.8 mM sodium citrate was added and the mixture was stirred until the color turned wine-red. The solution was cooled to room temperature and stored at 4 °C. The ∼13 nm sized AuNPs were functionalized with thiolated capture DNA probes (Cp_Hg–Ag_) as described previously.^38,47^ The resulting AuNP–Cp_Hg–Ag_ was purified using centrifugation and re-suspended in an equal volume of nuclease-free water. The nanoparticles were characterized using a Cary 60 UV-Vis Spectrophotometer (Agilent Technologies Inc., USA). For HCR experiments, the AuNP–Cp_Hg–Ag_ was re-suspended in sodium phosphate buffer (50 mM Na_2_HPO_4_/0.4 M NaNO_3_, pH 7.5) before use. Dynamic light scattering (DLS) measurements were recorded to determine the size of the nanoparticles before and after the HCR process using a DynaPro Titan (Wyatt technology Corporation, USA).

#### Programmable Hg^2+^ and/or Ag^+^ detection

All DNA concentrations were determined using a NanoDrop ND-1000 Spectrophotometer. 50 mM sodium phosphate buffer (Na_2_HPO_4_/0.4 M NaNO_3_, pH 7.5) was used for all DNA polymerization studies with nanoparticles. The 1 nM target metal ion (Hg^2+^ or Ag^+^), 1.0 μM initiator (I_Hg_ or I_Ag_) and 5.5 μM H1 & H2 concentrations were used in each programming study. Initially 1.0 μM of the initiator (I_Hg_ or I_Ag_) strand and 1 nM of a metal ion target were incubated with 200 μL of AuNP–Cp_Hg–Ag_ at 4 °C for 90 min. This nanoprobe assembly was centrifuged at 12 100 rpm until a clear supernatant was observed (∼10 min). The supernatant was discarded and the nanoparticle pellet was resuspended in the phosphate buffer. 5.50 μL of a 200 μM H1 & H2 stock solution was added to the nanoparticle assembly to the final concentration of 5.5 μM. The samples were incubated at 4 °C for 4 hours. The samples were then centrifuged at 12 100 rpm for 10 min and the nanoparticle pellet was resuspended in the phosphate buffer. Finally, 2.2 μL of 1 M of Mg(NO_3_)_2_ solution was added to 40 μL of the nanoparticle assembly to a final concentration of ∼55 mM Mg^2+^ in phosphate buffer. The color-transition was recorded 5, 45 or 60 min after the addition of Mg(NO_3_)_2_. UV-Vis spectra of each sample were recorded in 384 PCR well-plate (Applied Biosystems/VWR) using a BioTek Synergy microplate reader. Control experiments were performed in the absence of target metal ions, correct metal targets or initiators, and/or H1 & H2 hairpins.

#### Sensitivity measurements

Visual and spectroscopic detections were performed using various amounts (for visual: 0, 10 pM, 100 pM, 500 pM, 1 nM, 2 nM, 5 nM, and 10 nM; for spectroscopic: 0, 100 aM, 1 fM, 10 fM, 100 fM, 200 fM, 500 fM, 1 pM, 10 pM, 100 pM, 500 pM, 1 nM, 5 nM, and 10 nM) of target metal ion (Hg^2+^ or Ag^+^) in 40 μL of solution and the color-transitions were recorded 45 min after the addition of Mg(NO_3_)_2_. The change in the optical density (OD) at 520/700 nm (Abs_520/700_) of the resulting nanoparticle assembly was used to plot the aggregation rate and degree.

#### Selectivity measurements

The selectivity of the nanoprobe was evaluated by monitoring the response of the assay to a series of metal ions (nitrate salts), including 1 μM of Cd^2+^, Mn^2+^, Cu^2+^, Pb^2+^, Zn^2+^, Co^2+^, Ni^2+^, Ca^2+^, Mg^2+^ and a cocktail of all of these metal ions, where the Ca^2+^ and Mg^2+^ concentration was chosen to be 1 mM. In order to demonstrate the detection of Hg^2+^ or Ag^+^ with each metal ion and in the metal ion cocktail, 1 nM of Hg^2+^ or Ag^+^ was added to each sample. 200 μL of AuNP–Cp_Hg–Ag_, 1.0 μM initiator (I_Hg_ or I_Ag_) and 5.5 μM H1 & H2 were used for the study. The color-transitions were recorded 45 min after the addition of ∼55 mM Mg(NO_3_)_2_.

#### Detection in water

To test real samples with the proposed method, water samples (w_1_: tap water in UAlbany campus, w_2_: UAlbany Indian Pond, w_3_: Hudson River and w_4_: Rensselaer Lake) from regional sources were collected. All samples were filtered through a 0.22 μm syringe filter prior to the tests. The unspiked samples were used as is and the spiked samples were prepared by the addition of 50 μL of stock Hg^2+^ solution into the 450 μL water samples to a final concentration of 10 nM of Hg^2+^. 20 μL of these samples were added into 180 μL of sensor solution to a final concentration of 1.0 nM of Hg^2+^ (for the spiked sample). The experimental phosphate buffer prepared using ultrapure water was used as a reference. The remaining HCR steps were performed as described above.

#### Detection in soil

Soil samples were collected from the UAlbany campus. One gram of soil was mixed with 5 mL of ultrapure water, which was followed by a two-step filtration process using standard filter paper and a 0.22 μm syringe filter, respectively. For the spiked sample 10 nM of Hg^2+^ was added prior to the filtration process. 20 μL of the samples were added into 180 μL of sensor solution to a final concentration of 1.0 nM of Hg^2+^ (for the spiked sample). The experimental phosphate buffer prepared using ultrapure water was used as a reference. The remaining HCR steps were performed as described above.

#### Detection in urine

The unprocessed urine sample was used to illustrate the detection in biological fluids. Prior to the test, a fraction of the specimen (200 μL) was spiked with 10 nM of Hg^2+^. Both spiked and unspiked samples were centrifuged at 12 000 rpm at 4 °C for 20 min to eliminate any cell pellets or large biological content. The supernatant was collected from the top two thirds of the microtube container and used without further processing. 20 μL of samples were added into 180 μL of sensor solution to a final concentration of 1.0 nM of Hg^2+^ (for the spiked sample). The experimental phosphate buffer prepared using ultrapure water was used as a reference. The remaining HCR steps were performed as described above.

## Author contributions

MY conceived the study with MR and MB and designed the experiments. MR performed the nanoprobe characterization and detection experiments. MB helped with some of the sensitivity and selectivity studies. N. R., M. S. H. and S. Y. contributed intellectually to the experimental methods. M. Y. and M. R. wrote the manuscript.
